# Track Hub Quickload Translator: Convert Track Hub or Quickload data for viewing in the UCSC Genome Browser or the Integrated Genome Browser

**DOI:** 10.64898/2026.03.26.708838

**Published:** 2026-03-30

**Authors:** Nowlan Freese, Karthik Raveendran, Jaya Sravani Sirigineedi, Udaya Chinta, Philip Badzuh, Omkar Marne, Chirag Shetty, Irvin Naylor, Saideepthi Jagarapu, Ann E. Loraine

**Affiliations:** 1 Department of Bioinformatics and Genomics, University of North Carolina at Charlotte, Kannapolis, NC 28081, USA

## Abstract

**Summary:**

Track Hub Quickload Translator is a web application that interconverts University of California Santa Cruz (UCSC) Genome Browser track hub and Integrated Genome Browser (IGB) data repository formats by translating the track hub or Quickload configuration files to the other genome browsers required format. This new work enables researchers to work with tens of thousands of published genome assemblies for the first time using either browser.

**Availability and Implementation:**

Track Hub Quickload Translator is implemented using Python 3 and freely available to use at translate.bioviz.org. Integrated Genome Browser is available from BioViz.org. Track Hub Quickload Translator, GenArk Genomes, and the Integrated Genome Browser source code is available from github.org/lorainelab.

## Introduction

Genome browsers play a valuable role in visually analyzing aligned genomic data in detail. There are many different genome browsers, each with their own strengths, such as unique visual analytics tools or large collections of genome assemblies and annotations (Diesh, et al., 2023; Dyer, et al., 2025; Kent, et al., 2002; Nicol, et al., 2009; Robinson, et al., 2011). When users open any genome browser, they expect that either their studied genome assembly will be available, or the browser will be able to integrate a user provided genome assembly and aligned data. To aid users in organizing, configuring, and integrating large genomic datasets, the different genome browsers support distinct standards for data collections.

The University of California Santa Cruz (UCSC) Genome Browser is a web-based genome browser that provides access to thousands of genome assemblies and annotations (Casper, et al., 2026). Users can view and share their own data within the UCSC browser system using track hubs, a UCSC-specific data repository format (Raney, et al., 2014). To make a track hub, users deploy data files in Web-accessible locations alongside configuration files that define data track properties, such as color or initial visibility. The browser then uses the hub’s configuration files to retrieve and display the tracks alongside any UCSC-provided datasets for the same genome assemblies.

The Integrated Genome Browser (IGB) is a desktop genome browser with a focus on visual analytics (Freese, et al., 2016; Keim, et al., 2008). IGB visual analytics include color-coding data by numeric or categorical properties, filtering tracks to focus on a feature of interest, and creating new tracks from existing tracks using track operators. Users can install additional visualization features, such as user created filters and track operators, through IGB Apps (Shanbhag, et al., 2022).

IGB supports the Quickload format for organizing and distributing data for visualization. Like Track Hubs, Quickload uses metadata configuration files to specify track appearance and behavior. Quickloads can be stored locally for private use as well as on-line for public distribution using cyberinfrastructure such as CyVerse (Raveendran, et al., 2022; Swetnam, et al., 2024). The IGB team uses internet accessible Quickloads to provide commonly used genome assemblies and annotations to the browser.

Through either track hubs or Quickloads, users can organize and integrate their data collections for visualization. Previously, once a collection was set up as a track hub or a Quickload, it could only be viewed in its respective genome browser. This lack of interoperability adds additional complexity for users trying to view their own or other group’s data in their genome browser of choice.

To solve this problem for IGB and the UCSC Genome Browser, we have created Track Hub Quickload Translator, a Web application that interconverts Quickload and Track Hub metadata configuration formats, enabling visualization of the same data in both browsers. We demonstrate the value of this new work by presenting visual analysis of a human gene encoding mesenchyme homeobox protein 1, using both browsers to view, sanity-check and analyze experimental and computational results. Lastly, we present a new IGB App that uses the Track Hub Quickload Translator to make tens of thousands of genome assemblies and gene model annotations available within IGB, imported as Quickloads translated from GenArk project track hubs.

## Results

### Track Hub Quickload Translator web application

**Figure 1:**
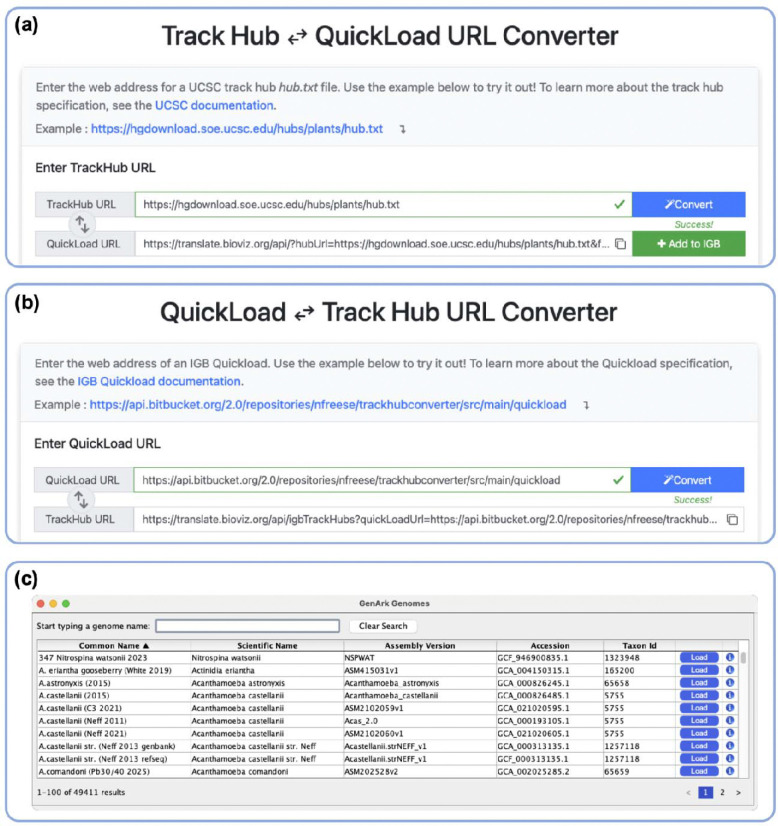
(a) User interface of the Track Hub Quickload Translator showing a UCSC track hub URL converted into an IGB Quickload URL. The Quickload can be added to a running instance of IGB by clicking on the Add to IGB button. (b) User interface of the Track Hub Quickload Translator showing an IGB Quickload URL converted into a track hub URL. The converted URL can be added as a track hub to the UCSC Genome Browser on the UCSC Connected Hubs page. (c) Snapshot of the GenArk Genomes app running in IGB showing the available assemblies that can be added to IGB from UCSC.

We deployed Track Hub Quickload Translator at translate.bioviz.org, where users can enter a web-accessible track hub URL and obtain a Quickload URL, or vice versa. Figure 1a shows an example URL translation workflow where a user has entered a track hub URL in the upper input field and clicked the “Convert” button. JavaScript loaded with the page has validated the input URL and then output an IGB Quickload URL that references a backend Application Programming Interface (API) endpoint from the translate.bioviz.org domain. This new URL is displayed to the user next to a button labeled “Add to IGB”. Clicking the button adds the new Quickload to IGB using a local endpoint implemented in IGB versions 9.1.10 and above.

Clicking on the double arrow button in the translate.bioviz.org interface switches the direction of conversion, so that a user can start with a Quickload URL and output a Track Hub URL (Fig. 1b). Once made, the new Track Hub URL can be added as a new data section within UCSC Genome Browser using the Connected Hubs tab of the Track Data Hubs page (“Track Data Hubs,” 2026).

Using the configuration settings included in the metadata files of both formats, data tracks from a track hub or a Quickload appear with close-to-correct formatting in both genome browsers. Within IGB, a track hub appears in the Available Data tab as a single folder. Opening the folder shows track names next to checkboxes for adding tracks to the IGB main view. Similarly, Quickload data loaded in the UCSC Genome Browser is organized into a Hub section where users can select tracks to load or change their display mode. Many of the data file formats are supported between the two browsers, including BAM, CRAM, VCF, bigBed, bigWig, and 2bit.

### Visual analysis of RNA-Seq samples

To demonstrate how Track Hub Quickload Translator could be used to visualize data in each genome browser, we investigated alternative splicing of a human gene encoding mesenchyme homeobox 1. By viewing RNA-Seq data from twenty human tissues in both browsers side-by-side, we found that an exon-skipped splice variant lacking a conserved DNA-binding domain was expressed in three tissue samples: heart, prostate and lung (Supplemental file 1.)

### GenArk Genomes IGB App

The UCSC Genome Browser team has created a collection of tens of thousands of genome assemblies and annotations called Genome Archive (GenArk) built as a track hub collection (Clawson, et al., 2023). Because the GenArk collection utilizes the track hub format, Track Hub Quickload Translator can be used to add any of the available genome assemblies to IGB. We created an IGB App called GenArk Genomes to facilitate adding these genome assemblies to IGB. The GenArk Genomes App can be installed from the IGB App Manager as of IGB version 10.2.0.

Once installed, GenArk Genomes appears as a new tabbed panel in IGB (Fig. 1c). The GenArk Genomes tab shows a searchable list of the nearly 50,000 genome assemblies available via GenArk. Users can search the assemblies by species common name, scientific name, assembly version, accession, or taxon id. When a user clicks the load button, the selected genome assembly track hub URL is translated into a Quickload URL via the Track Hub Quickload Translator backend and then added to IGB. The selected genome is then opened in IGB, and data tracks, such as gene annotations, appear in the Available Data window. From there users can load data and utilize IGB’s visual analytics tools as if the genome assembly were provided by an IGB Quickload site.

## Methods

Track Hub Quickload Translator is a web application implemented in Python v3.1 and the Django v3.2 web application framework. The application includes a user-facing frontend and a backend API that handles Hub to Quickload interconversion logic. The conversion logic works by converting Quickload to track hub formats, or vice versa, functioning as a façade interface between the formats (Gamma, et al., 1994).

The track hub format requires a track database file for the given assembly that specifies the location of each data file and their display properties. Likewise, the Quickload format requires an annots.xml file that specifies the location of each data file, how it is organized into a directory structure, and how it will be displayed in the genome browser. When a request to a converted URL for a track hub or Quickload is received by the Track Hub Quickload Translator back end, it reads the source format’s track configuration and then returns the translated property format expected by the target browser.

For the analysis of RNA expression data from twenty human tissues, we retrieved FastQ sequence files from the Sequence Read Archive BioProject PRJNA280600 and aligned them to reference human genome assembly hg38 using the splice-aware aligner TopHat2 (version 2.1.1), producing BAM (binary alignment) files. Scripts developed for the data processing are available at our git repository (github.com/lorainelab/srp056969). Data were then organized into a Track Hub and visually analyzed in Integrated Genome Browser version 10.2.0 and UCSC Genome Browser. Track Hub configuration files along with instructions for how to open the Track Hub in the UCSC Browser are available from bitbucket.org/nfreese/trackhub-human-hub.

## Discussion

Track Hub Quickload Translator makes it possible for genomic data stored in UCSC track hub or IGB Quickload collections to be viewed in either genome browser. These browsers offer distinct features and advantages and together can give users more integrated and detailed views of their data than either browser can achieve alone. By using a façade pattern to mask the complexity of parsing and reconfiguring the required metadata files, Track Hub Quickload Translator provides a single easy-to-use interface for users to translate their data collections.

## Supplementary Material

Supplement 1

Supplementary Data

Supplementary data is available online.

## Data Availability

Links to data can be found in hg38/trackDb.txt from https://bitbucket.org/nfreese/trackhub-human-hub.
