# Supplementary material for: Track Hub Quickload Translator: Convert Track Hub or Quickload data for viewing in the UCSC Genome Browser or the Integrated Genome Browser
Source: bioRxiv. 2026 Mar 30:2026.03.26.708838. Preprint. [Version 1] doi: 10.64898/2026.03.26.708838 (PMC13060283; doi:10.64898/2026.03.26.708838)
Supplement: Supplement 1 [file media-1.pdf]

Supplemental File 1

Introduction

This Supplemental File describes an example “use case” visual analysis demonstrating the Track Hub Quickload Translator Web application, together with the Integrated Genome Browser (IGB) and the UCSC Genome browser. For this example, we investigated alternative splicing of the human gene encoding mesenchyme homeobox 1 (*MEOX1*).

According to the National Center for Biotechnology Information (NCBI) Gene database, *MEOX1* is “a member of a subfamily of non-clustered, diverged, antennapedia-like homeobox-containing genes (Sayers, et al., 2025).” The Gene database record reports multiple distinct gene models, one of which skips a coding region exon. Loss of this second exon causes a frameshift in the third, downstream exon, deleting the protein’s DNA-binding region, a homeobox domain (Loraine, et al., 2003).

Loss of DNA-binding coding sequence in the exon-skipped variant suggests that this alternatively spliced transcript encodes an inactive, non-functional protein. However, if the skipped form were to be expressed at a high level, it could potentially play a negative, antagonistic role by sequestering *MEOX1* protein binding partners and interfering with non-exon-skipped *MEOX1* activity. Thus, to understand the function of the gene in regulating target gene expression, it is essential to determine the expression level of exon-skipped versus the exon-included variants.

Results

To investigate whether the exon-skipped variant is highly expressed relative to the exon-included form, we looked for evidence of each variants’ expression, using a publicly available RNA-Seq data set featuring sequences from twenty human tissues (BioProject PRJNA280600) (Duff, et al., 2015). We aligned the RNA-Seq sequence data to the hg38 reference human genome assembly, deployed the resulting output files to a Web-accessible location, and then created Track Hub configuration meta-data files that organize the data into a Track Hub. We deployed the Track Hub using free hosting within a Bitbucket git repository (https://bitbucket.org/nfreese/trackhub-human-hub). We then used the Track Hub, the Track Hub Quickload Translator Web application, the UCSC Genome Browser, and IGB to answer: Is the exon-skipped form highly or lowly expressed compared to the exon-included form?

Supplementary Figure 1a shows the Track Hub loaded into IGB using the Track Hub Quickload Translator. The track labeled “Heart_reads” comes from the Track Hub and shows RNA-Seq read alignments from heart tissue. The vertical dimension within the track shows fifteen distinct rows of individual alignments, with a top row showing a summary of the remaining alignments, too numerous to be shown in the available visible space. The track labeled “RefSeq Curated” shows three reference *MEOX1* gene models, each appearing as blocks linked in a series, where blocks represent exons and links represent introns removed during splicing. The “<” symbols superimposed on the intron connector lines indicate that transcription proceeds from right to left, on the minus strand of the reference assembly sequence.

To assess RNA-Seq evidence for the exon-skipping splicing pattern, we used two visual analytics operator functions in IGB: Depth Graph and Find Junctions (Gulledge, et al., 2014). The Depth Graph track operator facilitates assessing the pattern and number of all RNA-Seq alignments overlapping a region. It makes a new graph track in IGB showing the number of alignments from the source track that overlap each base position along the horizontal sequence axis. Find Junctions compares spliced RNA-Seq alignments within a selected track to each other and then creates a new track of exon-intron-exon features summarizing splicing evidence from the source track. Each exon-intron-feature is shown with a score indicating the number of alignments with gaps that perfectly match the feature’s intron boundaries.

The track labeled “Depth Graph” contains the Depth Graph operator output, a coverage graph indicating the number of alignments overlapping each base pair position. As is typical for RNA-Seq coverage graphs, the number of overlapping alignments varies across the gene body, as shown by the uneven peaks and valleys formed by the graph’s top edge. The coverage graph is tallest at the boundaries of exons, but we also see two small, low coverage regions of RNA-Seq alignments in the 5’ (right most) intron. Visualization of the heart sample compared to the other nineteen tissues showed that the heart RNA-Seq data contained more *MEOX1* alignments than the others (not shown). We hypothesized that the exon-skipped variant, if it is indeed produced in healthy tissue, would likely appear in this dataset derived from healthy heart tissue.

To investigate splicing differences, we used the Find Junctions track to quantify exon-exon junctions exposed by gapped sequence read alignments. The RefSeq Curated track shows three RefSeq-annotated gene models for *MEOX1*, with the NM_013999.3 gene model having the exon skipping event described above. Comparing the RefSeq Curated track to the Find Junctions track shows only four of the sequence read alignments from this dataset support the exon skipping event, indicating this is a rare splicing pattern in this sample. We also examined *MEOX1* splicing in all other tissue samples from this same dataset. We observed the exon-skipped variants in two other samples from the same collection of 20 samples (not shown). These were: prostate and lung. These results indicate that the exon-skipped form can occur in healthy human tissues and is expressed at lower levels than the fully functional, exon-included isoform in the twenty tissue types tested.

To further explore the role of splicing in *MEOX1* transcription and gene function, we used ProtAnnot, an app available from the IGB App Store (Mall, et al., 2016). ProtAnnot displays gene models differently than in IGB or other genome browsers, using fill color to indicate an exon’s frame of translation. By comparing exon colors vertically across stacked gene models, users can quickly notice potential frame-altering differences between splice variants. ProtAnnot also enables users to search for matches against the IntroPro compendium of conserved amino acid motif and display the results next to the gene models, showing which exons or parts of exons encode conserved motifs (Blum, et al., 2025). With ProtAnnot, users can quickly determine if or how splicing differences affect conserved, functional regions of corresponding protein products.

Previously, we used an early version of ProtAnnot to show that the skipped exon and the downstream third exon in *MEOX1* encodes a predicted homeodomain (Loraine, et al., 2003). For the current study, we used the latest versions of ProtAnnot and InterPro to re-evaluate the previous result. As before, ProtAnnot showed that exons 2 and 3 in the exon-included gene model match homeodomain motifs. Supplementary Figure 1b shows an image created using ProtAnnot, showing just the results of searching the Pfam protein motif database. In this view, ProtAnnot shows that exon-included gene models NM_004527.4 and NM_001040002.2 contain a homeodomain which is missing from the exon skipped form NM_013999.3. The difference in color for the final coding exon serves as a visual cue signaling how exon skipping causes a frameshift in that exon. This visualization confirms that the exon-skipped form encodes a protein that likely cannot bind DNA, whereas the exon-included form probably can because it encodes an intact homeodomain region.

(a)

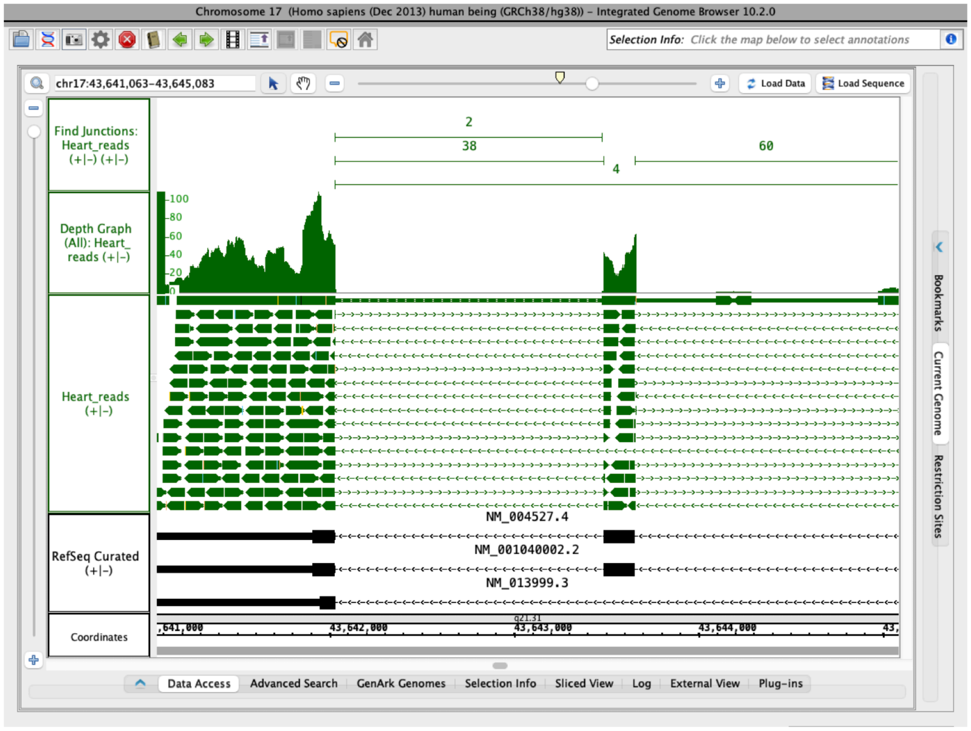

(b)

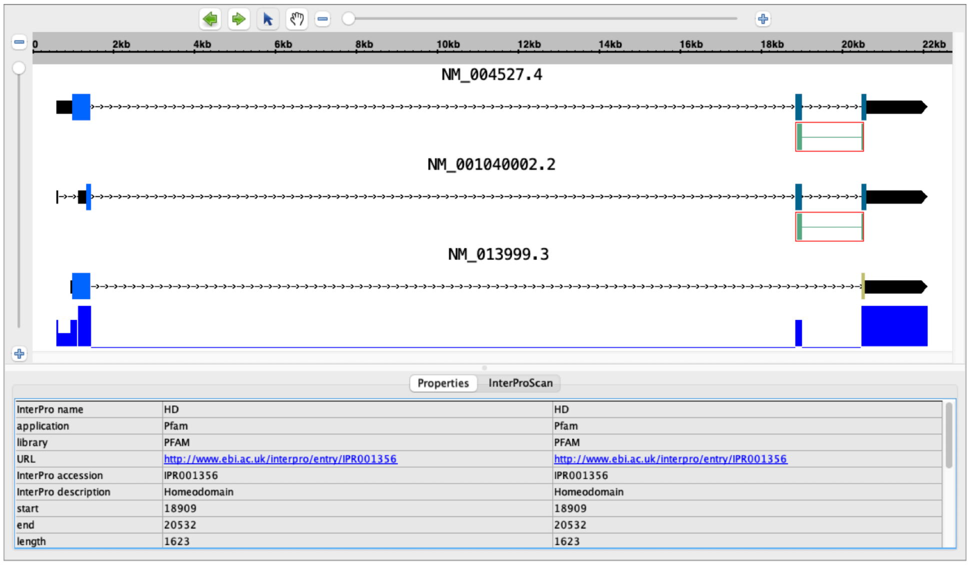

Figure S1. Example Track Hub loaded in the Integrated Genome Browser (IGB) showing RNA-Seq alignments from heart tissue to the gene *MEOX1*. (a) Integrated Genome Browser user interface displaying tracks from top down: Find Junctions track generated by IGB with counts of reads split by introns, Depth Graph generated by IGB displaying read counts, heart reads aligned to the hg38 genome, and *MEOX1* gene models. **(b)** ProtAnnot IGB app showing the *MEOX1* gene models and InterPro predicted protein motifs (green blocks highlighted in red box).

To gain further insight into alternative splicing of *MEOX1* we turned to the UCSC Genome Browser. The UCSC Genome Browser features over 37,000 data tracks for the human genome assemblies (GRCh38/hg38 and GRCh37/hg19), including many relevant to splicing regulation and alternative splicing (Perez, et al., 2025).

Supplementary Figure 2 shows the *MEOX1* gene region with heart RNA-Seq alignments from the same Track Hub viewed previously in Integrated Genome Browser. Three gene models appear at the top of the image, labeled with their RefSeq accessions, as in the IGB image. Similar to IGB, the UCSC Genome Browser shows the direction of transcription for minus strand genes like *MEOX1* as proceeding from right to left. In this new view, the aligned heart tissue reads from the Track Hub are shown with the track in fully expanded mode, where every aligned sequence is visible in maximum detail. This differs from the IGB image, which we configured to show fewer distinct alignments to save room for the track operator outputs. In this new view, each alignment is visible, and we can see there are exactly four alignments that support the exon-skipped form, confirming IGB's Find Junctions output.

From the many tracks available in the UCSC browser, we selected four relevant to splicing analysis: Spliced expressed sequence tags (ESTs), SpliceAI Acceptor Minus, SpliceAI Donor Minus, and AbSplice Scores tracks. These and other tracks can be added to the genome browser's current view by scrolling down the page and selecting menu or folder options that are organized into sections for different types or sources of data.

The Spliced ESTs track was available in the section labeled "RNA and Transcriptome." It contains alignments for human ESTs submitted to GenBank that contained evidence of at least one canonical intron (Benson, et al., 2013). Of the spliced ESTs that overlapped *MEOX1*, only EST (DA875966) supported the exon skipped variant. Clicking the DA875966 EST opened a popup with more information, revealing it was from prostate tissue (Kimura, et al., 2006). The other ESTs all supported the exon-included form and were from many different sources.

Next, we looked at the SpliceAI Acceptor Minus and SpliceAI Donor Minus tracks, which are included as part of the Splicing Impact super track, within a section titled "Phenotypes, Variants, and Literature". These tracks were created by SpliceAI, an open-source deep learning algorithm that predicts splicing probability (Jaganathan, et al., 2019). Peaks in the SpliceAI Acceptor Minus track at the 5' side of the exons in view indicate that they are computationally predicted to be acceptor sites, lining up with our RNA-Seq data. (Transcription proceeds from right to left for *MEOX1* in this view, so the left side of intron is a donor site, and the right side is an acceptor site.) In the SpliceAI Donor Minus track at the 3' of the second exon there is a peak that lines up with the gene model, but a second smaller donor peak is predicted downstream. Interestingly this alternate donor is supported by two reads in the IGB FindJunctions track and the reads can be seen within the aligned RNA-Seq data in the UCSC Genome Browser.

Lastly, we looked at the AbSplice Scores track, also part of the Splicing Impact super track. The AbSplice track shows predicted scores for aberrant splicing for all possible single-nucleotide variants (SNVs) across human tissues (Wagner, et al., 2023). At the level of detail shown, scored variants appear as color-coded marks, where warmer (red) colors represent higher scores. Higher scores represent an increased likelihood of aberrant splicing in at least one tissue. Again, looking at the second exon, we see a clustering of orange and red AbSplice scores around its donor and acceptor sites indicating a medium to high likelihood of aberrant splicing. Zooming in to select individual sites, we observed that the highest scored sites were for tissues that included heart atrial appendage and heart left ventricle.

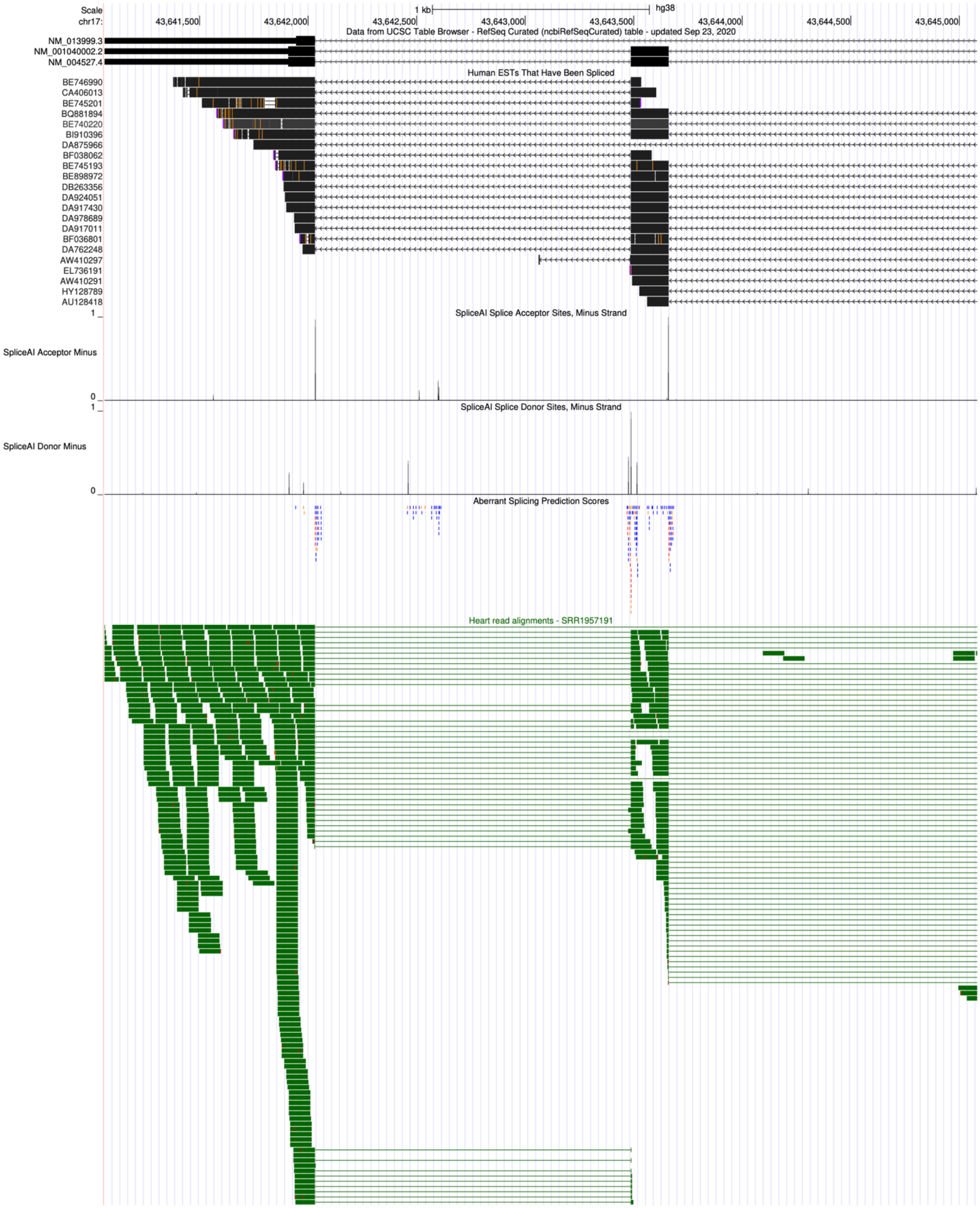

Figure S2. Example Track Hub loaded in the UCSC Genome Browser showing RNA-Seq alignments from heart tissue to the gene *MEOX1*. UCSC Genome Browser user interface displaying tracks from top down: *MEOX1* gene models, Spliced expressed sequence tags (ESTs), SpliceAI Acceptor Minus, SpliceAI Donor Minus, AbSplice Scores, and heart reads aligned to the hg38 genome.

Discussion

We used Integrated Genome Browser, the UCSC Genome Browser, a custom Track Hub we created for this study, and the Track Hub Quickload Translator to investigate alternative splicing of a 3-exon human gene encoding mesenchyme homeobox 1 (*MEOX1*). By viewing the same data in two browsers with different features, we observed the effects of splicing on gene function from previously published data. Using the IGB App ProtAnnot, we showed that exon-skipping deletes a DNA-binding homeobox domain encoded by the middle and third, final exons in *MEOX1*. We then examined newer data provided in our custom Track Hub, data files containing RNA-Seq alignments from twenty human tissue samples representing different tissues. We found that the exon-skipped variant was lowly-expressed compared to the non-skipped variant. This suggested that the exon-skipped form is expressed at low levels in wherever *MEOX1* transcripts are made, but it is still unknown whether the exon-skipped form has any biological activity.

We observed an intriguing coincidence having to do with tissue type. Of the twenty sample RNA-Seq datasets, the sample with the highest relative abundance for the exon-skipped variant was the prostate sample. When we viewed the spliced EST alignments dataset in the UCSC Browser, we saw that the one alignment that matched the exon-skipped form was also from a prostate sample. An interesting and important next step would be to analyze more RNA-Seq data from human prostate to determine if the high relative expression we observed in prostate mRNA was not simply an artifact of the one dataset we examined.

To identify possible splicing regulation in *MEOX1*, we investigated the output of SpliceAI using the UCSC Genome Browser, which provides a “Splicing Impact” super-track with scored annotations predicting effects of known genetic polymorphisms on splicing patterns. We observed that polymorphisms with high alternative splicing-inducing scores were clustered near a donor site flanking the middle exon, important for forming the U1 snRNP complex, an initial step in splicing reaction (Hoskins and Moore, 2012). Once formed, the donor-adjacent complex ultimately joins with a U2 snRNP downstream acceptor site complex, which removes the intervening sequence and ligates the flanking exons. SpliceAI’s identification of alternative splicing-including variations near the middle exon suggest possible competition between the middle-exons 3’ donor and the first exons’ 3’ donor. When the upstream donor “wins” the competition for the third exons acceptor, the exon-skipped variant can form. Discovery of sample types or tissues where the exon-skipped predominates could lead to mechanistic insights into splicing.

Supplementary References

Benson, D.A., *et al.* (2013) GenBank. *Nucleic Acids Res*;41(Database issue):D36-42.

Blum, M., *et al.* (2025) InterPro: the protein sequence classification resource in 2025. *Nucleic Acids Res*;53(D1):D444-d456.

Duff, M.O., *et al.* (2015) Genome-wide identification of zero nucleotide recursive splicing in Drosophila. *Nature*;521(7552):376-379.

Gulledge, A.A., *et al.* (2014) A protocol for visual analysis of alternative splicing in RNA-Seq data using integrated genome browser. *Methods Mol Biol*;1158:123-137.

Hoskins, A.A. and Moore, M.J. (2012) The spliceosome: a flexible, reversible macromolecular machine. *Trends Biochem Sci*;37(5):179-188.

Jaganathan, K., *et al.* (2019) Predicting Splicing from Primary Sequence with Deep Learning. *Cell*;176(3):535-548.e524.

Kimura, K., *et al.* (2006) Diversification of transcriptional modulation: large-scale identification and characterization of putative alternative promoters of human genes. *Genome Res*;16(1):55-65.

Loraine, A.E., *et al.* (2003) Exploring alternative transcript structure in the human genome using blocks and InterPro. *J Bioinform Comput Biol*;1(2):289-306.

Mall, T., *et al.* (2016) ProtAnnot: an App for Integrated Genome Browser to display how alternative splicing and transcription affect proteins. *Bioinformatics*;32(16):2499-2501.

Perez, G., *et al.* (2025) The UCSC Genome Browser database: 2025 update. *Nucleic Acids Res*;53(D1):D1243-d1249.

Sayers, E.W., *et al.* (2025) Database resources of the National Center for Biotechnology Information in 2025. *Nucleic Acids Res*;53(D1):D20-d29.

Wagner, N., *et al.* (2023) Aberrant splicing prediction across human tissues. *Nature Genetics*;55(5):861-870.
